# *In Silico* Exploration of Novel Tubulin Inhibitors: A Combination of Docking and Molecular Dynamics Simulations, Pharmacophore Modeling, and Virtual Screening

**DOI:** 10.1155/2022/4004068

**Published:** 2022-01-15

**Authors:** Farzin Hadizadeh, Razieh Ghodsi, Salimeh Mirzaei, Amirhossein Sahebkar

**Affiliations:** ^1^Department of Medicinal Chemistry, School of Pharmacy, Mashhad University of Medical Sciences, Mashhad, Iran; ^2^Biotechnology Research Center, Pharmaceutical Technology Institute, Mashhad University of Medical Sciences, Mashhad, Iran; ^3^Department of Medicinal Chemistry, Faculty of Pharmacy, Hormozgan University of Medical Sciences, Bandar Abbas, Iran; ^4^Applied Biomedical Research Center, Mashhad University of Medical Sciences, Mashhad, Iran; ^5^Department of Biotechnology, School of Pharmacy, Mashhad University of Medical Sciences, Mashhad, Iran

## Abstract

Microtubules play a critical role in mitosis and cell division and are regarded as an excellent target for anticancer therapy. Although microtubule-targeting agents have been widely used in the clinical treatment of different human cancers, their clinical application in cancer therapy is limited by both intrinsic and acquired drug resistance and adverse toxicities. In a previous work, we synthesized compound 9IV-c, ((E)-2-(3,4-dimethoxystyryl)-6,7,8-trimethoxy-N-(3,4,5-trimethoxyphenyl)quinoline-4-amine) that showed potent activity against multiple human tumor cell lines, by targeting spindle formation and/or the microtubule network. Accordingly, in this study, to identify potent tubulin inhibitors, at first, molecular docking and molecular dynamics studies of compound **9IV-c** were performed into the colchicine binding site of tubulin; then, a pharmacophore model of the 9IV-c-tubulin complex was generated. The pharmacophore model was then validated by Güner–Henry (GH) scoring methods and receiver operating characteristic (ROC) analysis. The IBScreen database was searched by using this pharmacophore model as a screening query. Finally, five retrieved compounds were selected for molecular docking studies. These efforts identified two compounds (**b** and **c**) as potent tubulin inhibitors. Investigation of pharmacokinetic properties of these compounds (**b** and **c**) and compound **9IV-c** displayed that ligand **b** has better drug characteristics compared to the other two ligands.

## 1. Introduction

Microtubules made of *α*- and *β*-tubulin heterodimers in eukaryotic cells and are vital components of the cytoskeleton which are involved in numerous cellular processes such as cell signaling, cell motility, and intracellular vesicle transport [1–3].

Microtubules form highly dynamics mitotic spindles, which are vital for the suitable orientation and segregation of chromosomes; disruption of this equilibrium will lead to cell cycle arrest or cell apoptosis [4–6].

Microtubule-targeting agents (MTAs) are divided into two classes including microtubule stabilizers such as taxanes which bind to the paclitaxel binding site that stabilize the tubulin polymer form and microtubule-destabilizing agents like vinca alkaloids and colchicine which bind to the colchicine and vinblastine binding site which inhibit tubulin polymerization into microtubules [6–8]. Despite the progress in the administration of microtubule targeting agents for the treatment of patients with cancer, currently, there are no FDA-approved tubulin inhibitors targeting the colchicine binding site [9]. This has encouraged medicinal chemists to design and discover the novel antimitotic agents that bind to the colchicine binding site for cancer therapy [10–16].

Pharmacophore is defined as an ensemble of steric and electronic features that is necessary to reach the optimal interactions of a ligand with the catalytic site of a protein and very well accepted in the medicinal chemistry laboratory [17–19].

**Figure 1:**
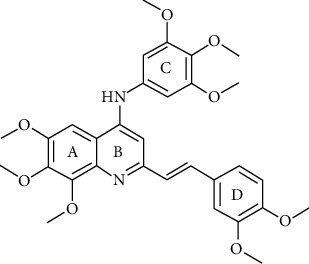
Chemical structure of compound 9IV-c.

In a previous work, we synthesized a new series of styrylquinolines that among them, compound 9IV-c, ((E)-2-(3,4-dimethoxystyryl)-6,7,8-trimethoxy-N-(3,4,5-trimethoxyphenyl)quinoline-4-amine) (Figure 1) exhibited a potent activity against multiple human tumor cell lines, by targeting spindle formation and/or the microtubule network [4]. In this work, to identify potent tubulin inhibitors, at first, to explain the possible binding mode of compound 9IV-c and to evaluate the validity of docking results, molecular docking and molecular dynamics studies of this compound, respectively, were performed into the colchicine binding site of tubulin; then, a pharmacophore model of 9IV-c-tubulin complex was generated. It was then used as a screening query to obtain potent small molecules from an IBScreen database. Five hits were obtained in this step for a match to the pharmacophore model which was subjected to molecular docking studies. Finally, two compounds **b** and **c** with the lowest binding-free energies were selected as potent tubulin inhibitors. The pharmacokinetic properties of these compounds were studied using SwissADME software.

## 2. Results and Discussion

### 2.1. Molecular Modeling

**Figure 2:**
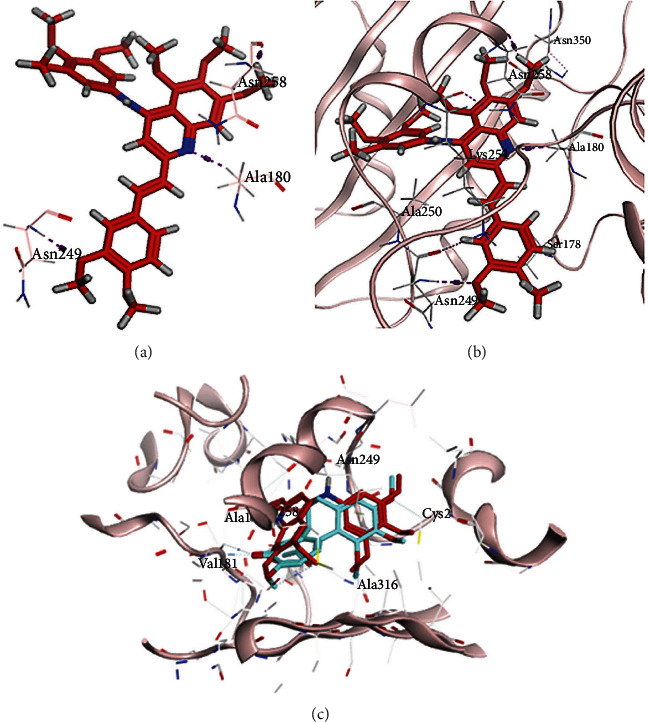
(a) Hydrogen bonding of **9ІV-c** with colchicine binding site of tubulin (purple dashed line). (b) Binding pose and hydrophobic interactions of **9ІV-c** with tubulin. (c) Comparison of the docking mode of **9ІV-c** (red) and colchicine (blue) in active site of colchicine in tubulin.

To study the manner of interactions between compound **9ІV-c** and tubulin, this compound was docked into the colchicine binding site of tubulin (PDB ID: 4O2B) via MOE 2015.10 [4]. The studies exhibited that the compound **9ІV-c** docked well in the colchicine-binding site with a binding free energy of -9.759 kcal/mol (Figures 2(a) and 2(b)). The hydrogen-bonding interactions (purple dashed line in Figure 2(a)) were seen between the methoxy group of A-ring of compound **9ІV-c** with residue Asn 258*β* and also nitrogen atom of quinoline ring with Ala 180*α* and the methoxy group of D-ring with Asn 249*β*. Further, the hydrophobic interactions between quinolines with different amino acids such as Gln11*α*, Asn258*β*, Thr352*β*, Lys254*β*, Ser178*α*, Ala250*β*, Asn350*β*, Val351*β*, Lys352*β*, and Leu255*β* have been observed (Figure 2(b)).

Figure 2(c) shows the binding mode of compound **9ІV-c** with colchicine, the standard tubulin inhibitor, at the active site of colchicine in tubulin. The binding mode observed for **9ІV-c** was very similar to that for colchicine in the cocrystallized tubulin structure. The trimethoxyphenyl group of **9ІV-c,** which is an essential pharmacophore for tubulin inhibitors, was placed in the same position as the corresponding moiety of colchicine.

### 2.2. Molecular Dynamics Simulation

Molecular dynamics (MD) simulation studies were performed to obtain a more exact ligand-receptor model in a state near to the natural situations [7]. For this goal, the docked structure of compound **9ІV-c** was used as the initial structure for 100 ns MD simulations. After accomplishment of the simulation process, the complex was examined for root mean square deviation (RMSD), radius of gyration (Rg), and hydrogen bonds [20].

**Figure 3:**
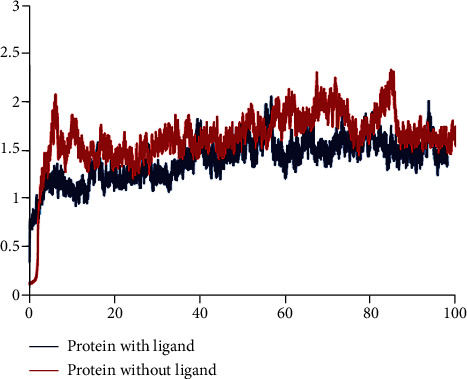
RMSD between protein with ligand and without ligand.

RMSD determines the stability of the constructions. The RMSD of backbone (C*α*, C, and N) of apo-form and ligand-bond protein was shown in Figure 3. The average RMSD value of ligand-bond protein was 1.536 ± 0.212 Å, whereas the apo-form was with an average RMSD value of 1.776 ± 0.292 Å. In RMSD of apo-form, the change was larger than ligand-bond protein. It seemed that binding of the ligand with protein decreased the conformation flexibility of protein and the complex reached stability during the simulation.

**Figure 4:**
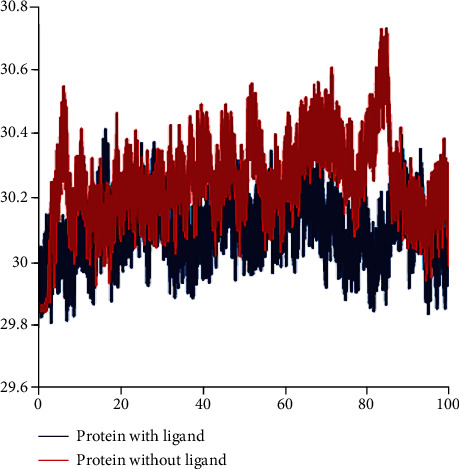
Rg of protein with ligand and without ligand.

Rg (readius of gyration) is an indicator of the protein structure density and measures the distance of region's parts from its center of gravity. As shown in Figure 4. Rg values of the apo-form and ligand-bond protein were 30.24 ± 0.125 and 30.089 ± 0.0.091 Å (mean ± SD), respectively. This confirms the stabilization and nonsignificant conformational changes in the structure of the ligand-bound protein [20].

**Figure 5:**
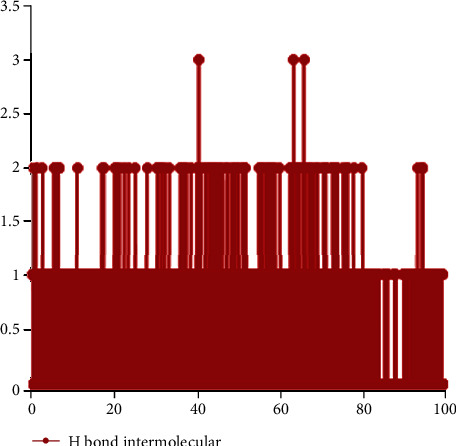
Number of hydrogen bonds between protein and ligand in time scale.

**Figure 6:**
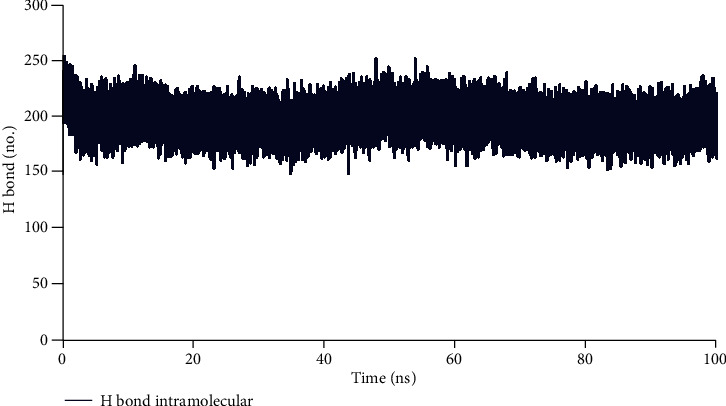
Number of intramolecular hydrogen bonds of protein in time scale.

Hydrogen bonds have a key role in the stability of the ligand-protein complex. This stability of the hydrogen-bond network was because of the presence of the ligand in the protein binding site. The findings revealed that ligand made one to two hydrogen bonds with protein (Figure 5). These hydrogen bonds increased binding affinity of the ligand with protein. The mean of intramolecular hydrogen bonds of the protein was 196.96 ± 11.96 (Figure 6.). These hydrogen bonds stabilized the secondary structure of protein.

**Figure 7:**
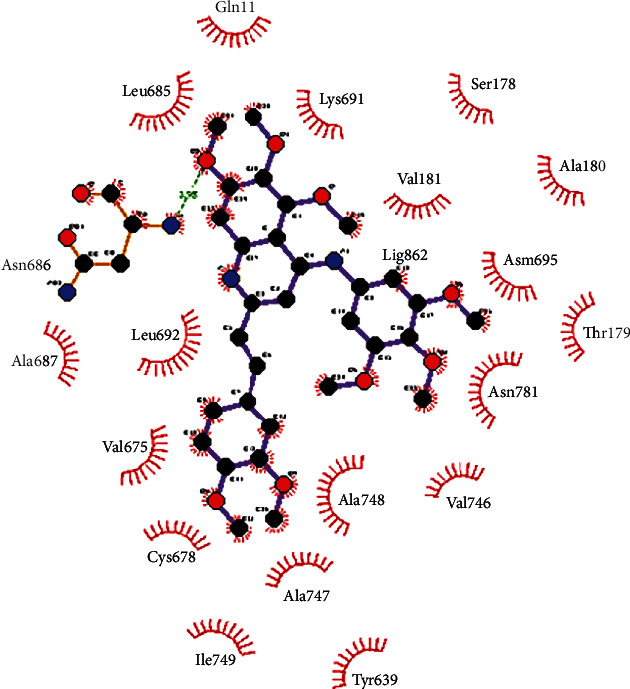
A diagrammatic representation of interactions between ligand **9ІV-c** at the active site of tubulin after 100 ns molecular dynamics simulation. The green dotted lines represent hydrogen bond interaction, and red arcs with radiating spokes represent the amino acids showing hydrophobic interaction with protein. Carbon, nitrogen, and oxygen atoms have been shown in black, blue, and red, respectively. The violet lines represent the ligand bond.

The 2D representation of the interaction between compound **9ІV-c** with the colchicine binding site after 100 ns simulation by LigPlot has been depicted in Figure 7.

### 2.3. Pharmacophore Generation

**Figure 8:**
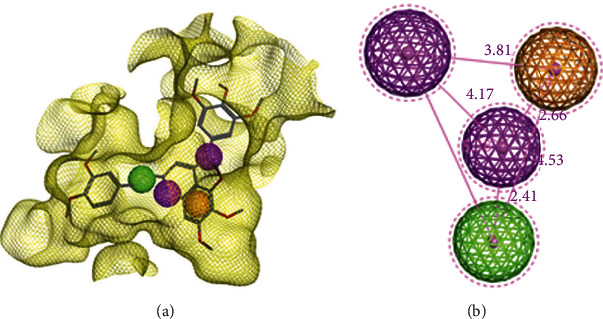
The generated pharmacophore model in the colchicine binding site of tubulin. (a) Pharmacophore features are color-coded: purple, two hydrogen bond donor features (F1& F2: Don); orange, one aromatic feature (F3: Aro); green, one hydrophobic feature (F4: Hyd). (b) 3D spatial relationship and distance between pharmacophore features (Å).

Molecular docking and molecular dynamics studies showed that the compound **9ІV-c** binds well to the colchicine binding site of tubulin and the interaction of this ligand with the tubulin, stabilizes the protein structure. Therefore, using MOE software, a pharmacophore model was generated based on the docked structure of compound **9ІV-c** in the colchicine binding site, to acquire the chemical features on the inhibitor binding of the tubulin [18, 21]. The generated model included four features (Figures 8(a) and 8(b)): two hydrogen bond donor feature (F1 & F2: Don) which are generated by nitrogen atom of quinoline ring and the nitrogen atom of aniline ring, one aromatic feature (F3: Aro), and one hydrophobic feature (F4: Hyd) which are produced by a double bond of styryl section.

### 2.4. Validation of the Pharmacophore Model

To validate the reliability of the pharmacophore model constructed in this study, Güner–Henry (GH) scoring methods and receiver operating characteristic (ROC) analysis were used.

#### 2.4.1. GH Scoring Method

The GH score method was used to evaluate the discriminative ability of the pharmacophore model in distinguishing active compounds from the inactive compounds from a testing set database including 20 known tubulin inhibitors and 600 inactive molecules [3, 12, 22–25]. The GH analysis by computing parameters such as total hits (Ht), active hits (Ha), enrichment factor (*E*), and goodness of hit score (GH) were performed (Table 1). GH score was observed to be 0.69 for the model, representing that the pharmacophore model has a good ability to discriminate active compounds from inactive ones.

#### 2.4.2. ROC Studies

**Figure 9:**
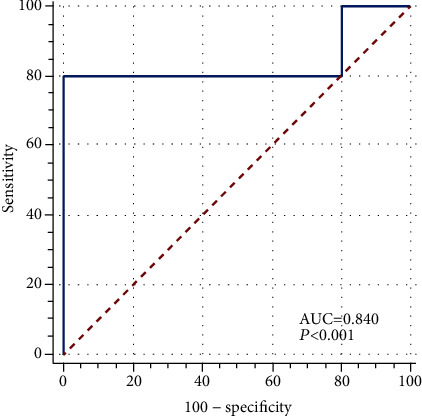
Receiver operating characteristic (ROC) curves of pharmacophore models.

The pharmacophore model was used to receiver operating curve (ROC) analysis to assess its ability to suitably classify a list of compounds as active or inactive [26]. The ROC curve obtained for the validation showed an AUC value of 0.84 (Figure 9), indicating that the model differentiated the active compounds from the inactive ones efficiently (*p* < 0.001). The sensitivity and specificity of the model were 80% and 98.66%, respectively.

### 2.5. Virtual Screening and Identifying Potential Inhibitors

The IBScreen database (containing 158210 compounds) was searched by using the 2D chemical structure of compound **9ІV-c** (Figure 1.) as a query to find compounds with similar structures to this compound [27]. At this step, a total of 1257 compounds were obtained based on the most similarity to compound **9ІV-c**; this set was then screened based on the created pharmacophore model. As a result, five ligands (a–e) were confirmed to fit with the pharmacophore model (Table 2). These compounds were then docked into the colchicine binding site, and two ligands **b** and **c** were selected with the lowest binding-free energies and the best modes of interactions with the colchicine binding site.

**Figure 10:**
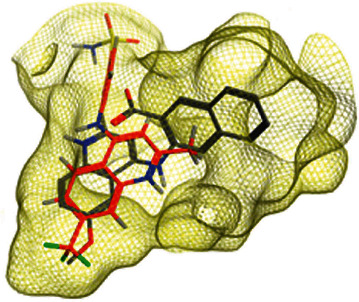
The superimposed structure of compounds **b** (in gray) and **c** (in red) into the colchicine binding site of tubulin.

**Figure 11:**
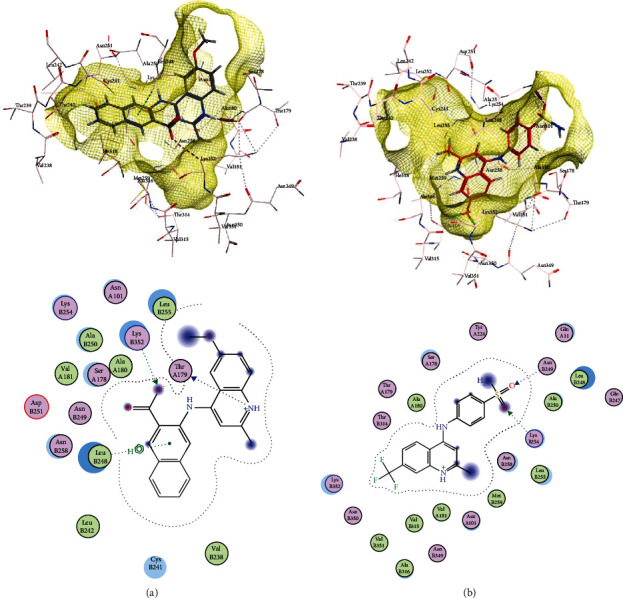
Two- and three-dimensional representation of compounds (a) **b** and (b) **c** in colchicine binding site of tubulin.

Interactions of compounds **b** and **c** with tubulin protein are shown in Figures 10 and 11. It can be observed that compounds **b** and **c** have better free binding energies in comparison with compound **9ІV-c** (-10.498 and -10.083 kcal/mol for **b** and **c**, respectively, and -9.759 for **9ІV-c**). This subject suggests that these compounds (**b** and **c**) may interact with higher potencies than compound **9ІV-c**.

As seen in Figure 11(a) and 11(b), molecular docking analysis of compound **b** displayed two hydrogen bond interactions between acidic substituents with residues of Lys 352*β* and the nitrogen atom of quinoline ring with residues of Thr 179*α* and a cation-*π* interaction between residue Leu 248*β* and one of the phenyl rings (Figure 11(a)). The sulfonami group of compound **c** formed two hydrogen bond interactions with residues Lys254*β* and Asn249*β* (Figure 11(b)). These compounds formed hydrophobic interactions with residues Leu255*β*, Lys248*β*, Asn258*β*, Ser178*α* and Asn101*α*, and Gln11*α*.

### 2.6. Pharmacokinetic Property Prediction

In this study, by using the SwissADME software, drug development involves the process of adsorption, distribution, metabolism, and excretion (ADME), for three ligands **b**, **c,** and **9ІV-c** was performed [28]. The test results can be seen in Table 3.

The results of the tests in the gastrointestinal absorption parameter showed that ligand **b** (with the high result) are suitable for oral use. It was also found that ligands **c** and **9ІV-c** with low results are not well used orally, but may be used as an injection.

The results of yes, in the cytochrome inhibitor parameter, will indicate that ligand can act as an inhibitor in the process of cytochrome metabolism, while no results will show that the ligand cannot act as an inhibitor in the process of cytochrome metabolism [29]. The test results show that three ligands were cytochrome inhibitors.

The higher bioavailability of the compound indicates that it can be better used orally [29]. Based on the results obtained from this parameter (Table 3), all three ligands have good bioavailability values, and among these, ligand **b,** which exhibited better results in docking studies, has better bioavailability.

## 3. Conclusion

In this research, molecular docking and molecular dynamics studies of compound **9IV-c** (a potent inhibitor of tubulin polymerization found in previous work) were performed into the colchicine binding site of tubulin. The studies exhibited modes of interaction of compound **9ІV-c** with tubulin including the formation of hydrogen bonds and hydrophobic interactions and also characterized the stability of this compound during a 100 ns simulation run. Then, a pharmacophore model of the 9IV-c-tubulin complex was generated. The created model included two hydrogen bond donor features (F1 & F2: Don), one aromatic feature (F3: Aro), and one hydrophobic feature (F4: Hyd). The pharmacophore model was then validated by Güner–Henry (GH) scoring methods and receiver operating characteristic (ROC) analysis. GH score was observed to be 0.69 for the model, representing that the pharmacophore model has a good ability to discriminate active compounds from inactive ones.

The IBScreen database (containing 158210 compounds) was searched by using the 2D chemical structure of compound **9ІV-c** as a query to find compounds with similar structures to this compound and was then screened based on the generated pharmacophore model, and five ligands (a–e) verified to fit with the pharmacophore model. These compounds were then docked into the colchicine binding site, and two ligands **b** and **c** were selected with the lowest binding free energies and the best modes of interactions with the colchicine binding site. These ligands (ligands **b** and **c)** and compound **9ІV-c** were tested for ADMET parameters and pharmacokinetic properties. The results displayed that ligand **b** has better drug characteristics compared to the other two ligands.

## 4. Materials and Methods

### 4.1. Molecular Modeling

The 2D structure of the compound was organized in ChemDraw Ultra 8.0 software, and the 3D structure was obtained using Hyperchem 7 software through molecular mechanic force filed preoptimization and then by AM1 semiempirical calculation. The X-ray crystal structure of tubulin (PDB ID: 4O2B) was downloaded from the PDB (Protein Data Bank). Further changes (addition of polar hydrogen or deletion of water molecules) were done by MOE software. Compounds were docked into the binding site of tubulin using MOE software. The top-score docking poses were selected for final ligand-target interaction analysis using the LigX module in MOE Software [30].

### 4.2. Molecular Dynamics Simulation

Molecular dynamics (MD) simulation studies were performed to find the interaction between protein and ligand in atomic details in the real natural situation (aqueous solution at *T* = 37°C, *P* = 1 atm). Calculations were performed using NAMD 2.12 program (http://www.ks.uiuc.edu/Research/namd) with the CHARMM27 force field. To analyze results Visual Molecular Dynamics (VMD) (http://www.ks.uiuc.edu/Research/VMD) was used. The force field parameters of ligand **9ІV-c** were provided by SwissParam (http://swissparam.chr). The whole systems were immersed in the center of a TIP3 water box with dimensions 97.117 Å × 113.789 Å × 97.287 Å, using the VMD program. The systems were neutralized by adding sodium and chloride ions. The Particle-Mesh Ewald (PME) algorithm with a grid spacing of 1 Å and periodic boundary conditions was applied. A cut-off of 15 (Å) was used for the short-range Lennard-Jones interactions. Finally, MD simulations were performed with a time step of 2 fs for 100 ns. The trajectory of the system was stored at every 1 ps and analyzed by VMD analyzer tools. The system was complex and required significant computational. A system with a processor: AMD Ryzen Threadripper 1950X 16- Core, 3.40 GHz and installed memory (RAM) 32.0 GB, was used in this study [31].

### 4.3. Pharmacophore Model Generation

The docked structure of compound 9IV-c in the colchicine binding site of tubulin was used for pharmacophore model generation. To create the most descriptive features of the colchicine binding site of tubulin, the Pharmacophore Query Editor of MOE was employed. These features are indicated as spheres that characterize the important interaction points of the ligand with the active site of tubulin [18, 29].

### 4.4. Validation of the Pharmacophore Model

#### 4.4.1. GH Scoring Method

The Güner–Henry (GH) scoring method quantifies the importance of the generated model by retrieving the active compounds from a database containing known active and inactive molecules [23, 32]. To evaluate the discriminating ability of the generated pharmacophore model a testing set database including 20 known tubulin inhibitors with experimental activity and 600 inactive molecules was searched by the pharmacophore model as a 3D structural search query. The retrieved compounds are selected based on according to their RMSD value. Lower RMSD values show better quality of the matching of the molecule to the pharmacophore model. The GH score was calculated using the following formulae: [24].

(1) $$\begin{array}{c} \text{EF}=\frac{H_{a}/H_{t}}{A/D},\\ \text{GH}=\frac{H_{a}\;\left(3\;A+H_{t}\right)}{4H_{t}\;A}\left(1-\frac{H_{t}-H_{a}}{D-A}\right). \end{array}$$

Here, *D* is the number of compounds in the database, *A* is the number of active compounds, *H*_*t*_ is the number of hits retrieved, *H*_*a*_ is the number of actives in hit list, EF is the enrichment factor, and GH is the Güner–Henry score. The GH score ranging from 0.6 to 1 would indicate an optimal pharmacophore model.

#### 4.4.2. Analysis Using ROC

To evaluate the pharmacophore model, receiver operating characteristic (ROC) curve analysis was also performed using MedCalc statistical software (http://www.medcalc.org). In ROC analysis, the ability of the obtained pharmacophore model was indicated with the area under the curve (AUC) to distinguish a list of compounds as active or inactive compounds in terms of two parameters, sensitivity and specificity.

### 4.5. Screening of the Database Based on Pharmacophore Models

The IBScreen database containing 158210 compounds was used. At first, using ChemFinder software the 2D chemical structure (SMILES file format) of compound **9ІV-c** (Figure 1.) was used as a query in the IBScreen database to find compounds with similar structures to this compound. The applied filter gave a total of 1257 compounds. The result structures were downloaded in a SDF format. Database preparation in MOE software was performed based on default settings. Then, the generated pharmacophore model (3D structure) was used as a screening query by MOE to find compounds with similar binding features of compound 9IV-c as tubulin inhibitor. Compounds retrieved from pharmacophore filtering were then docked into the colchicine binding site of tubulin, and the ligands with the lowest docking energies were chosen.

## Figures and Tables

**Table 1:** Pharmacophore model evaluation based on the Güner–Henry scoring method.

Serial no.	Parameter	Pharmacophore model
1	Total molecules in database (*D*)	620
2	Total number of active in database (*A*)	20
3	Total hits (Ht)	24
4	Active hits (Ha or Tp)	16
5	% yield of actives ((Ha/Ht) × 100)	66.66
6	% ratio of actives ((*H*_*a*_/*A*) × 100)	80
7	Enrichment factor (*E*) ((*H*_*a*_ × *D*)/(*H*_*t*_ × *A*))	20.66
8	False negatives (*A* − *H*_*a*_)	4
9	False positives (*H*_*t*_ − *H*_*a*_)	8
10	True negative	592
11	Se^a^ %	80
12	Sp^b^%	98.66
13	Goodness of hit score	0.69

^a^Se: sensitivity (TP/TP + FN); ^b^Sp: specificity (TN/TN + FP).

**Table 2:** Two-dimensional representations of compounds retrieved from the IBScreen database.

Compounds	IBScreen ID	Structures	Binding energies (kcal/mol)
a	STOCK4S-95238	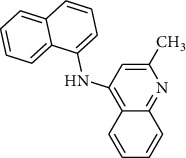	-7.842
b	STOCK1S-15990	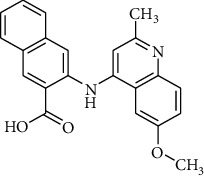	-10.498
c	STOCK6S-14477	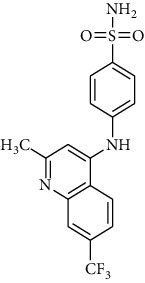	-10.083
d	STOCK3S-48351	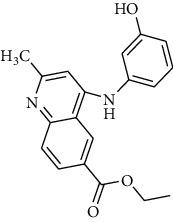	-8.014
e	STOCKIS-43560	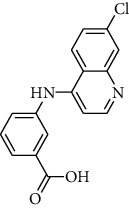	-8.350

**Table 3:** The pharmacokinetic properties prediction by SwissADME software.

Ligand	GI absorption			Cytochrome inhibitor			Bioavailability
		1	2	3	4	5	
b	High	Yes	Yes	Yes	Yes	No	0.85
c	Low	Yes	Yes	No	Yes	Yes	0.55
9ІV-c	Low	NO	Yes	Yes	No	No	0.55

1: CYP1A2; 2: CYP2C19; 3: CYP2C9; 4: CYP2D6; 5: CYP3A4.

## Data Availability

Data are available upon a reasonable request from the corresponding author.

## References

[1] Li L., Jiang S., Li X., Liu Y., Su J., Chen J. (2018). Recent advances in trimethoxyphenyl (TMP) based tubulin inhibitors targeting the colchicine binding site. *European Journal of Medicinal Chemistry*.

[2] Lai Q., Wang Y., Wang R. (2018). Design, synthesis and biological evaluation of a novel tubulin inhibitor ∗∗7a3∗∗ targeting the colchicine binding site. *European Journal of Medicinal Chemistry*.

[3] Sun Y., Pandit B., Chettiar S. N. (2013). Design, synthesis and biological studies of novel tubulin inhibitors. *Bioorganic & Medicinal Chemistry Letters*.

[4] Mirzaei S., Eisvand F., Hadizadeh F., Mosaffa F., Ghasemi A., Ghodsi R. (2020). Design, synthesis and biological evaluation of novel 5,6,7-trimethoxy- *N* -aryl-2-styrylquinolin-4-amines as potential anticancer agents and tubulin polymerization inhibitors. *Bioorganic Chemistry*.

[5] Zhou Y., Yan W., Cao D. (2017). Design, synthesis and biological evaluation of 4-anilinoquinoline derivatives as novel potent tubulin depolymerization agents. *European Journal of Medicinal Chemistry*.

[6] Beckers T., Reissmann T., Schmidt M. (2002). 2-Aroylindoles, a novel class of potent, orally active small molecule tubulin inhibitors. *Cancer Research*.

[7] Mirzaei S., Hadizadeh F., Eisvand F., Mosaffa F., Ghodsi R. (2020). Synthesis, structure-activity relationship and molecular docking studies of novel quinoline-chalcone hybrids as potential anticancer agents and tubulin inhibitors. *Journal of Molecular Structure*.

[8] Nakagawa-Goto K., Taniguchi Y., Watanabe Y. (2016). Triethylated chromones with substituted naphthalenes as tubulin inhibitors. *Bioorganic & Medicinal Chemistry*.

[9] Mirzaei S., Eisvand F., Hadizadeh F., Mosaffa F., Ghodsi R. (2020). Design, synthesis, and biological evaluation of novel 5, 6, 7-trimethoxy quinolines as potential anticancer agents and tubulin polymerization inhibitors. *Iranian Journal of Basic Medical Sciences*.

[10] Mirzaei S., Qayumov M., Gangi F., Behravan J., Ghodsi R. (2020). Synthesis and biological evaluation of oxazinonaphthalene-3-one derivatives as potential anticancer agents and tubulin inhibitors. *Iranian Journal of Basic Medical Sciences*.

[11] Behbahani F. S., Tabeshpour J., Mirzaei S. (2019). Synthesis and biological evaluation of novel benzo [c] acridine-diones as potential anticancer agents and tubulin polymerization inhibitors. *Archiv der Pharmazie*.

[12] Guggilapu S. D., Guntuku L., Reddy T. S. (2017). Synthesis of thiazole linked indolyl-3-glyoxylamide derivatives as tubulin polymerization inhibitors. *European Journal of Medicinal Chemistry*.

[13] Tantak M. P., Klingler L., Arun V., Kumar A., Sadana R., Kumar D. (2017). Design and synthesis of bis(indolyl)ketohydrazide-hydrazones: Identification of potent and selective novel tubulin inhibitors. *European Journal of Medicinal Chemistry*.

[14] Kumar A., Kumar M., Sharma S., Guru S. K., Bhushan S., Shah B. A. (2015). Design and synthesis of a new class of cryptophycins based tubulin inhibitors. *European Journal of Medicinal Chemistry*.

[15] Naret T., Bignon J., Bernadat G. (2018). A fluorine scan of a tubulin polymerization inhibitor isocombretastatin A-4: design, synthesis, molecular modelling, and biological evaluation. *European Journal of Medicinal Chemistry*.

[16] Sultana F., Shaik S. P., Nayak V. L. (2017). Design, synthesis and biological evaluation of 2-anilinopyridyl-linked oxindole conjugates as potent tubulin polymerisation inhibitors. *ChemistrySelect*.

[17] Sun H. (2008). Pharmacophore-based virtual screening. *Current Medicinal Chemistry*.

[18] Zhou Y., Tang S., Chen T., Niu M. M. (2019). Structure-based pharmacophore modeling, virtual screening, molecular docking and biological evaluation for identification of potential poly (ADP-ribose) polymerase-1 (PARP-1) inhibitors. *Molecules*.

[19] Markt P., Schuster D., Langer T. (2011). Pharmacophore models for virtual screening. *Virtual Screening: Principles, Challenges, and Practical Guidelines*.

[20] Aryapour H., Dehdab M., Sohraby F., Bargahi A. (2017). Prediction of new chromene-based inhibitors of tubulin using structure-based virtual screening and molecular dynamics simulation methods. *Computational Biology and Chemistry*.

[21] Tian Y. S., Kawashita N., Arai Y., Okamoto K., Takagi T. (2015). Pharmacophore modeling and molecular docking studies of potential inhibitors to E6 PBM–PDZ from human papilloma virus (HPV). *Bioinformation*.

[22] la Regina G., Bai R., Coluccia A. (2018). New 6- and 7-heterocyclyl-1 *H* -indole derivatives as potent tubulin assembly and cancer cell growth inhibitors. *European Journal of Medicinal Chemistry*.

[23] Li R.-J., Wang Y. L., Wang Q. H., Wang J., Cheng M. S. (2015). In silico design of human IMPDH inhibitors using pharmacophore mapping and molecular docking approaches. *Computational and Mathematical Methods in Medicine*.

[24] Lu S.-H., Hwang Y. C., Liu I. J. (2020). Development of therapeutic antibodies for the treatment of diseases. *Journal of Biomedical Science*.

[25] Zhao S., Li X., Peng W. (2021). Ligand-based pharmacophore modeling, virtual screening and biological evaluation to identify novel TGR5 agonists. *RSC Advances*.

[26] Gupta N., Sitwala N., Patel K. (2014). Pharmacophore modelling, validation, 3D virtual screening, docking, design and in silico ADMET simulation study of histone deacetylase class-1 inhibitors. *Medicinal Chemistry Research*.

[27] Hashemi S., Sharifi A., Zareei S., Mohamedi G., Biglar M., Amanlou M. (2020). Discovery of direct inhibitor of KRAS oncogenic protein by natural products: a combination of pharmacophore search, molecular docking, and molecular dynamics studies. *Research in Pharmaceutical Sciences*.

[28] Hecht D., Fogel G. B. (2009). Computational intelligence methods for docking scores. *Current Computer-Aided Drug Design*.

[29] Ekawati M. M., Nasution M. A. F., Siregar S., Rizki I. F., Tambunan U. S. F. Pharmacophore-based virtual screening and molecular docking simulation of terpenoid compounds as the inhibitor of sonic hedgehog protein for colorectal cancer therapy.

[30] Malayeri S. O., Abnous K., Arab A. (2017). Design, synthesis and biological evaluation of 7-(aryl)-2,3-dihydro-[1,4]dioxino[2,3- *g* ]quinoline derivatives as potential Hsp90 inhibitors and anticancer agents. *Bioorganic & Medicinal Chemistry*.

[31] Abdizadeh T., Kalani M. R., Abnous K. (2017). Design, synthesis and biological evaluation of novel coumarin-based benzamides as potent histone deacetylase inhibitors and anticancer agents. *European Journal of Medicinal Chemistry*.

[32] Ooms D., Palm R., Leemans V., Destain M. F. (2010). A sorting optimization curve with quality and yield requirements. *Pattern Recognition Letters*.

